# Checklist of the Braulidae, Camillidae, Diastatidae and Drosophilidae of Finland (Diptera, Ephydroidea)

**DOI:** 10.3897/zookeys.441.7057

**Published:** 2014-09-19

**Authors:** Jere Kahanpää

**Affiliations:** 1 Finnish Museum of Natural History, Zoology Unit, P.O. Box 17, FI–00014 University of Helsinki, Finland

**Keywords:** Checklist, Finland, Diptera, biodiversity, faunistics

## Abstract

A checklist of the Braulidae (1 species), Camillidae (3 spp.), Diastatidae (10 spp.) and Drosophilidae (67 spp.) of Finland is presented. Campichoetinae is included as a subfamily of Diastatidae.

## Introduction

The Ephydroidea is now recognized as a well-supported, probably monophyletic clade of acalyptrate flies (Wiegmann et al. 2011), although the correct placement of some of the included families – especially the very unsual bee louses of family Braulidae – is still being discussed. The relationships of the families within the superfamily are less understood and the families are listed alphabetically in the checklist below. Seven or eight families are currently included in Ephydroidea. Five of them are found in Finland. Four families are covered in this paper; the fifth (Ephydridae itself) has its own treatment in this volume of ZooKeys. The families missing from Finland are the Curtonotidae, Cryptochetidae, and Nannodastiidae with uncertain affinities (Papp and Mathis 2001). Griffiths (1972) proposed a new family, Campichoetidae, for *Campichoeta* Macquart, 1835. We follow the world catalogue of Diastatidae (Mathis and Barraclough 2011) and keep Campichoetinae as a subfamily of Diastatidae.

World catalogues have been recently published for Diastatidae (Mathis and Barraclough 2011) and Drosophilidae (Brake and Bächli 2008). The North European Drosophilidae were treated in detail by Bächli et al. (2004) and the review of Finnish *Drosophila* by Hackman (1954) is now obsolete. The Finnish species of Braulidae and Camillidae were last listed by Hackman (1980); the diastatids were treated by Chandler (1987).

**Table 1.** Number of species by family.

Family	Number of species in			Level of knowledge
	World (Pape et al. 2011)	Europe	Finland	
Braulidae	7	3	1	poor
Camillidae	40	8	3	average
Diastatidae	49 (Mathis and Barraclough 2011)	16	10	good
Drosophilidae	4003	121	67	good

## Checklist

suborder Brachycera Macquart, 1834

clade Eremoneura Lameere, 1906

clade Cyclorrhapha Brauer, 1863

infraorder Schizophora Becher, 1882

clade Muscaria Enderlein, 1936

parvorder Acalyptratae Macquart, 1835

superfamily Ephydroidea Zetterstedt, 1837

**BRAULIDAE** Egger, 1853

***BRAULA*** Nitzsch, 1818

*Braula coeca* Nitzsch, 1818

**CAMILLIDAE** Frey, 1921

***CAMILLA*** Haliday, 1838

*Camilla atrimana* Strobl, 1910

= *atripes* Duda, 1934

= *acutipennis* misid.

*Camilla flavicauda* Duda, 1922

= *glabrata* Collin, 1956

*Camilla glabra* (Fallén, 1823)

= *subfuscipes* Collin, 1933

**DIASTATIDAE** Hendel, 1917

CAMPICHOETINAE Griffiths, 1972

***CAMPICHOETA*** Macquart, 1835

*Campichoeta griseola* (Zetterstedt, 1855)

*Campichoeta obscuripennis* (Meigen, 1830)

DIASTATINAE Hendel, 1917

***DIASTATA*** Meigen, 1830

*Diastata adusta* Meigen, 1830

= *unipunctata* Zetterstedt, 1847

*Diastata boreonigra* Chandler, 1987

*Diastata costata* Meigen, 1830

*Diastata flavicosta* Chandler, 1987

*Diastata fuscula* (Fallén, 1820)

*Diastata nebulosa* (Fallén, 1823)

*Diastata ornata* Meigen, 1830

*Diastata vagans* Loew, 1864

**DROSOPHILIDAE** Rondani, 1856

STEGANINAE Hendel, 1917

***AMIOTA*** Loew, 1862

**sg. *Amiota*** Loew, 1862

*Amiota albilabris* (Roth in Zetterstedt, 1860)

*Amiota alboguttata* (Wahlberg, 1839)

*Amiota rufescens* (Oldenberg, 1914)

*Amiota subtusradiata* Duda, 1934

***CACOXENUS*** Loew, 1858

**sg. *Paracacoxenus*** Hardy & Wheeler, 1960

*Cacoxenus argyreator* Frey, 1932

***LEUCOPHENGA*** Mik, 1886

**sg. *Neoleucophenga*** Oldenberg, 1915

*Leucophenga quinquemaculata* Strobl, 1893

***PHORTICA*** Schiner, 1862

**sg. *Phortica*** Schiner, 1862

*Phortica variegata* Fallén, 1823

***STEGANA*** Meigen, 1830

**sg. *Stegana*** Meigen, 1830

*Stegana furta* (Linnaeus, 1766)

= *curvipennis* (Fallén, 1823)

**sg. *Steganina*** Wheeler, 1960

*Stegana baechlii* Laštovka & Máca, 1982

*Stegana coleoptrata* (Scopoli, 1763)

*Stegana hypoleuca* Meigen, 1830

*Stegana longifibula* Takada, 1968

*Stegana mehadiae* Duda, 1924

*Stegana nigrithorax* Strobl, 1898

*Stegana similis* Laštovka & Máca, 1982

DROSOPHILINAE Rondani, 1856

***CHYMOMYZA*** Czerny, 1903

*Chymomyza amoena* (Loew, 1862)

*Chymomyza caudatula* Oldenberg, 1914

*Chymomyza costata* (Zetterstedt, 1838)

*Chymomyza distincta* (Egger, 1862)

*Chymomyza fuscimana* (Zetterstedt, 1838)

***DROSOPHILA*** Fallén, 1823

**sg. *Dorsilopha*** Sturtevant, 1942

*Drosophila busckii* Coquillett, 1901

**sg. *Drosophila*** Fallén, 1823

*Drosophila ezoana* Takada & Okada, 1957

*Drosophila funebris* (Fabricius, 1787)

*Drosophila histrio* Meigen, 1830

*Drosophila hydei* Sturtevant, 1921

= *repleta* misid.

*Drosophila immigrans* Sturtevant, 1921

= *tripunctata* Becker, 1908 preocc.

*Drosophila limbata* von Roser, 1840

*Drosophila littoralis* Meigen, 1830

*Drosophila lummei* Hackman, 1972

*Drosophila montana* Stone, Griffen & Patterson, 1941

= *ovivororum* Lakovaara & Hackman, 1973

*Drosophila phalerata* Meigen, 1830

*Drosophila picta* Zetterstedt, 1847

*Drosophila repleta* Wollaston, 1858

*Drosophila subarctica* Hackman, 1969

*Drosophila testacea* von Roser, 1840

*Drosophila transversa* Fallén, 1823

*Drosophila vireni* Bächli, Vilela & Haring, 2002

**sg. *Sophophora*** Sturtevant, 1942

*Drosophila alpina* Burla, 1948

*Drosophila ambigua* Pomini, 1940

*Drosophila bifasciata* Pomini, 1940

*Drosophila eskoi* Lakovaara & Lankinen, 1974

*Drosophila ingrica* Hackman, 1957

*Drosophila melanogaster* Meigen, 1830

*Drosophila obscura* Fallén, 1823

*Drosophila simulans* Sturtevant, 1919

*Drosophila subobscura* Collin, 1936

*Drosophila subsilvestris* Hardy & Kaneshiro, 1968

= *silvestris* Basden, 1954 preocc.

*Drosophila tristis* Fallén, 1823

***HIRTODROSOPHILA*** Duda, 1924

*Hirtodrosophila cameraria* (Haliday, 1833)

*Hirtodrosophila confusa* (Staeger, 1844)

*Hirtodrosophila lundstroemi* (Duda, 1935)

*Hirtodrosophila oldenbergi* (Duda, 1924)

*Hirtodrosophila trivittata* (Strobl, 1893)

***LORDIPHOSA*** Basden, 1961

*Lordiphosa fenestrarum* (Fallén, 1823)

*Lordiphosa nigricolor* (Strobl, 1898)

***MICRODROSOPHILA*** Malloch, 1921

**sg. *Microdrosophila*** Malloch, 1921

*Microdrosophila congesta* (Zetterstedt, 1847)

**sg. *Oxystyloptera*** Duda, 1924

*Microdrosophila zetterstedti* Wheeler, 1959

= *nigriventris* (Zetterstedt, 1847) preocc.

***SCAPTODROSOPHILA*** Duda, 1923

*Scaptodrosophila deflexa* (Duda, 1924)

= *guyenoti* (Burla, 1948)

***SCAPTOMYZA*** Hardy, 1849

**sg. *Hemiscaptomyza*** Hackman, 1959

*Scaptomyza trochanterata* Collin, 1953

*Scaptomyza unipunctum* (Zetterstedt, 1847)

**sg. *Parascaptomyza*** Duda, 1924

*Scaptomyza pallida* (Zetterstedt, 1847)

= *disticha* (Duda, 1921)

**sg. *Scaptomyza*** Hardy, 1849

*Scaptomyza consimilis* Hackman, 1955

*Scaptomyza flava* (Fallén, 1823)

= *flaveola* (Meigen, 1830)

= *apicalis* Hardy, 1849

*Scaptomyza graminum* (Fallén, 1823)

*Scaptomyza griseola* (Zetterstedt, 1847)

*Scaptomyza montana* Wheeler, 1949

*Scaptomyza teinoptera* Hackman, 1955

## Excluded species

*Stegana strobli* Mik, 1898 misidentification

*Lordiphosa hexasticha* (Papp, 1971) not found within present borders
